# Phylogeny-aware Simulations Suggest a Low Impact of Unsampled Lineages in the Inference of Gene Flow During Eukaryogenesis

**DOI:** 10.1093/gbe/evaf190

**Published:** 2025-10-09

**Authors:** Moisès Bernabeu, Saioa Manzano-Morales, Toni Gabaldón

**Affiliations:** Barcelona Supercomputing Centre (BSC-CNS), Plaça Eusebi Güell, Barcelona 08034, Spain; The Barcelona Institute of Science and Technology, Institute for Research in Biomedicine (IRB Barcelona), Barcelona 08028, Spain; Barcelona Supercomputing Centre (BSC-CNS), Plaça Eusebi Güell, Barcelona 08034, Spain; The Barcelona Institute of Science and Technology, Institute for Research in Biomedicine (IRB Barcelona), Barcelona 08028, Spain; Barcelona Supercomputing Centre (BSC-CNS), Plaça Eusebi Güell, Barcelona 08034, Spain; The Barcelona Institute of Science and Technology, Institute for Research in Biomedicine (IRB Barcelona), Barcelona 08028, Spain; Catalan Institution for Research and Advanced Studies (ICREA), Barcelona, Spain; Centro de Investigación Biomédica En Red de Enfermedades Infecciosas (CIBERINFEC), Barcelona, Spain

**Keywords:** eukaryogenesis, phylogenomics, ghost lineages, horizontal gene transfer

## Abstract

The topologies of gene trees are broadly used to infer horizontal gene transfer events and characterize the potential donor and acceptor partners. Additionally, ratios between branch lengths in the gene tree can inform about the timing of transfers relative to each other. Using this approach, recent studies have proposed a relative chronology of gene acquisitions in the lineage leading to the last eukaryotic common ancestor. However, a recognized caveat of the branch-length ratio method are potential biases due to incomplete taxon sampling resulting in so-called “ghost” lineages. Here, we assessed the effect of ghost lineages on the inference of the relative ordering of gene acquisition events during eukaryogenesis. For this, we used a novel simulation framework that populates a dated Tree of Life with plausible “ghost” lineages and simulates their gene transfers to the lineage leading to last eukaryotic common ancestor. Our simulations suggest that a substantial majority of gene acquisitions from distinct ghost donors are inferred with the correct relative order. However, we identify phylogenetic placements where ghost lineages would be more likely to produce misleading results. Overall, our approach offers valuable guidance for the interpretation of future work on eukaryogenesis, and can be readily adapted to other evolutionary scenarios.

Significance Phylogenomic analyses are sensitive to missing data, such as incomplete taxon sampling (so-called “ghost” lineages). A reconstruction of the relative order of gene acquisitions in complex ancestral scenarios such as the origin of eukaryotes can therefore be affected by the available sampling of prokaryotic lineages. In this work, we leverage simulations to test for the robustness of inferred relative timing of gene transfers in the lineage leading to the ancestor of eukaryotes. Using two alternative dated topologies of the Tree of Life, we reveal a lower effect of ghost lineages in the qualitative conclusions than previously estimated using random species topologies. Our work not only clarifies the impact of unsampled lineages in the inference of eukaryotic origins but also outlines a methodological framework to study the impact of missing lineages in other ancestral scenarios.

## Introduction

Inferring a timeline for the origin and diversification of life on Earth stands as a major goal in evolutionary biology. Molecular dating performed on extant genetic sequences provides a means to achieve this. This approach relies on the idea of a correlation between time and sequence divergence, which is represented by the length of branches in a gene phylogeny (Zuckerkandl and Pauling 1965). Specifically, this divergence is the result of the combined effect of the time separating the two nodes delimiting a branch, and the evolutionary rate of that gene during that period. Molecular dating typically employs sets of orthologous genes with relatively straightforward evolutionary histories, often in combination with fossil data to establish absolute divergence times. This approach has successfully estimated divergence times for numerous lineages across the Tree of Life (TOL) (Strassert et al. 2021; Davín et al. 2025).

However, the timeline of events leading to eukaryotes (eukaryogenesis) remains unresolved due to challenges in applying molecular dating methods. Firstly, the microbial fossil record is sparse and often difficult to classify. Secondly, while the last eukaryotic common ancestor (LECA) and its divergence time from its closest archaeal relatives can be estimated (Moody et al. 2024), a significant time gap exists between these two ancestors, which actually encompasses the crucial transformative events such as organelle origins and mitochondrial endosymbiosis (Gabaldón 2021). Thirdly, many such events involve evolutionary processes like gene duplication or horizontal gene transfer (HGT) that are neither datable in the fossil record nor represented by the single-copy ortholog sets typically used in molecular dating.

**Fig. 1.**
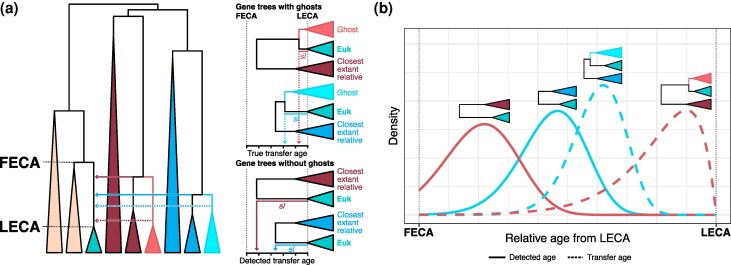
Schematic view of the effect of ghost lineages in the inference of relative timing of gene acquisitions. a) The tree on the left represents a simplified species tree containing eukaryotes (in green, whose MRCA is indicated as LECA), archaea (in beige, whose MRCA with eukaryotes is indicated as FECA), and a pair of bacterial donor lineages (in red and blue). Lighter colors represent the ghost lineages that actually donated the genes, whereas darker colors represent the closest sampled relatives from which inferences can be made. Dotted lines show the true transfer ages and the full line represents the inferred ages in the absence of ghosts in the gene trees. Trees in the right represent gene trees from which stem lengths are calculated, in presence and absence of ghosts, and the *x*-axis displays the inferred age. sl stands for “stem length”, the length of the branch from the last common ancestor of the eukaryotic sequences (i.e. the LECA node) and its MRCA with the closest extant prokaryotic sister. See Fig. S1 for additional detail. b) Overview of the branch-length ratio method. The waves represent the distributions of normalized stem lengths across gene trees for each donor. Shorter stem lengths correspond to more recent events. Distributions are shown for the inferred ages in full lines and the “true” transfer ages with dotted lines.

To circumvent these limitations and elucidate key evolutionary events in eukaryogenesis, Pittis and Gabaldón proposed the “branch-length ratio” method to estimate relative time differences between comparable events identified in different gene trees (Pittis and Gabaldón 2016a). This method compares branch lengths of interest across gene trees after normalizing them using the median branch length of a shared tree node that represents the same evolutionary time. Consequently, differences in these normalized lengths can reveal relative time differences between the examined branches. This allows for the comparison of the relative order in which, for instance, two gene acquisitions occurred within a given lineage (Fig. 1), and has been used, among other studies, to reconstruct the relative timing of gene acquisition and duplication events during eukaryogenesis (Pittis and Gabaldón 2016a; Vosseberg et al. 2021; Bernabeu et al. 2024b). This enables inferences about the contributions of lineages outside Asgard archaea (the ancestor of the nuclear lineage) and alphaproteobacteria (the ancestor of mitochondria), as well as the timing of mitochondrial acquisition relative to the appearance of other defining characteristics of eukaryotic cells. The applicability of this approach was assessed and shown to include the conditions used in the original 2016 study (Susko et al. 2021).

The branch-length ratio method has prompted some methodological debates (Pittis and Gabaldón 2016b; William et al. 2017; Susko et al. 2021; Tricou et al. 2022; Bernabeu et al. 2024a). One recent criticism focused on the potential for unsampled or extinct lineages (so-called “ghost lineages”) to mislead phylogenetic analyses, particularly concerning the inference of the relative order of HGT events (Tricou et al. 2022). Using a simulation framework, these authors demonstrated that, under certain conditions, the presence of ghost lineages can lead to inverted conclusions (a “shift”) regarding the relative order of acquisitions—for example, inferring that gene acquisition from lineage B preceded acquisition from lineage A, when the actual order was A before B. Nevertheless, their simulations also indicated that, across all simulated conditions, correct inferences (i.e. when the correct ordering is inferred) were always more probable than shifts.

However, as previously discussed (Bernabeu et al. 2024a; Tricou et al. 2024), these simulations presented certain limitations, most notably the use of simulated topologies entirely disconnected from current knowledge of the TOL and the relevant lineages in eukaryogenesis. This disconnect hinders the assessment of whether the conditions under which these shifts occur are plausible. Anchoring simulations to the existing understanding of the TOL allows for more realistic inferences regarding key parameters, such as the temporal window of relevant transfer events and the number and identity of extant lineages to which confounding ghost lineages might be related. To achieve this, we developed a new simulation framework that is grounded in two recently reconstructed and dated TOLs (Mahendrarajah et al. 2023; Moody et al. 2024) and explicitly models the transfer of genes from any potential ghost lineage branching from currently known bacterial lineages. Consequently, our model can not only assess the impact of ghost lineages in a specific phylogenomic analysis but also identify the influence of alternative topologies and highlight particularly problematic lineages or transfer scenarios. Our findings demonstrate overall lower probabilities of shift compared to previous simulation studies. Furthermore, our analysis indicates that previously established orderings of gene transfers in eukaryogenesis are robust to the presence of ghost lineages (Pittis and Gabaldón 2016a; Vosseberg et al. 2021). Beyond the study of gene transfer in the context of eukaryogenesis, the simulation framework presented here holds the potential for broader application in evaluating the impact of ghost lineages across diverse evolutionary questions.

## Results

To specifically evaluate the potential effect of plausible ghost lineages in previously inferred ordering of transfer events during eukaryogenesis (Pittis and Gabaldón 2016a), and to circumvent identified shortcomings of previous simulation-based assessments (Tricou et al. 2022; Bernabeu et al. 2024a), we developed a new simulation framework (see Methods and Fig. 3). As compared to previous attempts, this simulation framework introduces two key novel aspects. Firstly, instead of simulating tree topologies with a given birth or death ratio, it uses a reference dated TOL as a phylogenetic background. Secondly, instead of randomly pruning branches from a simulated tree, ghost lineages are specifically modeled by randomly populating the given reference TOL with new (ghost) lineages branching out from ancestral lineages that co-existed in the studied eukaryogenesis period, that is, the time spanning from the divergence of the closest archaeal relatives of eukaryotes (for simplicity, we refer to this node as FECA, the first eukaryotic common ancestor), to the LECA node (Gabaldón 2021). Finally, we simulate transfers from these ghosts at randomly chosen times from the birth of the ghost lineage to the origin of LECA.

Applying this framework to a recently dated TOL based on ATPase sequences (ATPase tree hereafter, adapted from Mahendrarajah et al. (2023)), we simulated 1 million pairs of ghost lineages, each undergoing a gene transfer event within a defined temporal sequence. Subsequently, we inferred the detected relative order of these transfers as when using the branch-length ratio method (Pittis and Gabaldón 2016a), conducted in the absence of the simulated ghost lineages (see Methods). We then quantified the proportion of instances where the inferred order of transfers was reversed compared to the simulated (true) order (i.e. shifts). Additionally, we calculated the proportion of shifts where one of the inferred transfer events predated FECA (Fig. S1). This is pertinent because inferred transfers older than FECA are typically excluded by studies such as Pittis and Gabaldón (2016a) due to the evident artifactual nature of such temporal inferences, as events associated with eukaryogenesis cannot logically precede the divergence of eukaryotes from their closest archaeal relatives, the Asgard archaea.

**Fig. 2.**
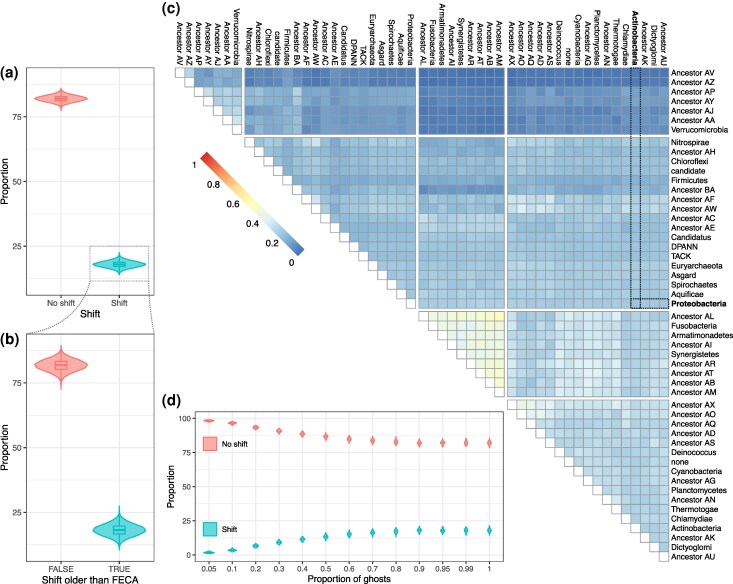
Proportion of shifted conclusions. a) Distributions for the proportion of shifts observed between pairs of simulated ghosts (left). b) For the shift-inducing cases, proportion of those that show an inferred distance older than FECA (outside FECA–LECA period). c) Proportion of shifts per pair of lineages, that is, the proportion of simulations resulting in a shift in transfers from the specified pair ancestors. Dashed lines show the relationships of Proteobacteria and Actinobacteria, in bold. The ancestors and their descendant phyla are associated in Table S1. d) Proportion of shifts observed between pairs of simulated transfers for a given proportion of ghost lineages. The transfer donors are inferred to be or not a ghost using a Bernoulli distribution with the probability parameter specified in the *x*-axis.

Our results indicate a relatively low overall fraction of shifts, around 18% (Fig. 2a). This suggests that around 18% of the examined gene tree pairs would lead to incorrect inferences regarding the relative order of transfer events. Conversely, 82% of gene tree pairs yielded the correct relative order, despite the absence of the actual donor lineages (“ghosts”). Furthermore, of the 18% of shifts observed, roughly 18% resulted in inferred transfer times falling outside the temporal window between FECA and LECA (Fig. 2b), and would therefore be disregarded in the context of eukaryogenesis. This translates to approximately 15% (82% of 18%) of gene tree pairs that are truly confounding. This percentage of error in the reconstruction of ancient events should be considered in light of other recognized sources of phylogenetic uncertainty (Steenwyk et al. 2023).

**Fig. 3.**
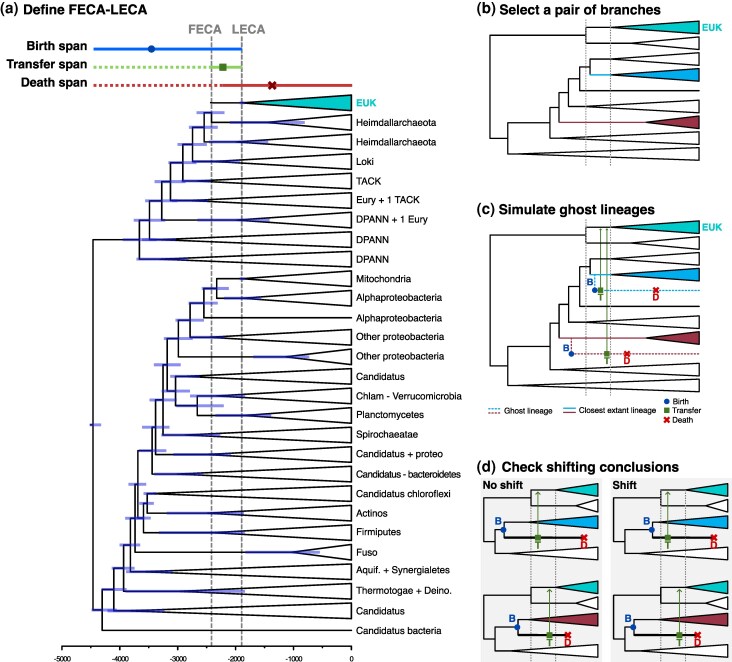
Simulation framework for inferring transfers to the FECA–LECA branch using a dated TOL. First, FECA and LECA are placed in the species tree (a) which defines the time window in which the simulation will occur and the lineages that will play a part, then a pair of lineages are selected (b) and ghosts are simulated on these branches, which will then transfer genes to the eukaryotic lineage (c). Finally, the difference between the true order of events and the one inferred from the gene trees (where the ghost lineages are not available and inferences are made from their closest sister) is assessed to see if the relative order is reverted (shift) or not (d).

To assess the effect of the underlying tree topology, we repeated the simulations employing an alternative dated TOL with sparser taxonomic sampling (hereafter referred to as the LUCA tree taken from Moody et al. (2024), Fig. S2). In this alternative scenario, the results are overall consistent with our primary analysis. Although the amount of shifted conclusions is slightly higher (21% on average, Fig. S2a), there is a higher proportion of shifts resulting in transfer times older than FECA (45%), leading to 12% (55% of 21%) of truly confounding trees (Fig. S2b). These differences may be attributable to the lower taxonomic sampling of the LUCA tree with respect to the ATPase tree (Fig. S3a, b). This sparser sampling appears to lead to older inferred birth times for ghost lineages and older transfers (Fig. S3c), which in turn results in a larger proportion of shifted simulations falling outside the FECA–LECA interval. Importantly, the distributions of distances between the ghost lineage birth (representing the inferred transfer age in the absence of the ghost) and the actual transfer age are similar, suggesting that the simulation process itself is not the primary driver of this difference, but rather the tree topology and its taxonomic sampling (Fig. S3d).

An additional advantage of grounding the simulations on a dated TOL is that it allows stratifying the likelihood of shift according to the phylogenetic position of the ghost, thereby pinpointing problematic clades or assessing the likelihood of wrong inference in previously proposed LECA donors. We assessed the likelihood of shifts for different lineages and their most recent common ancestors (MRCA), for which we assigned placeholder names (Table S1). Our results (Fig. 2c) show that some pairs of phyla are more likely to cause shifts than others. Particularly, simulations where ghosts originated from lineages Fusobacteria, Armatimonadetes, Synergistetes, and several ancestors of bacterial phyla like Ancestors AL, AI, AR, AT, AB, AM (see Table S1) resulted in a higher proportion of shifts when compared to other donor pairs, and are particularly problematic when paired with each other. These groups, entailing a higher risk to produce misleading inference when transfers originated from ghost lineages should be taken into account for further analyses of gene flow during eukaryogenesis. Nevertheless, these particular clades are not discussed as potential significant gene donors in previous studies using the branch-length ratio method (Pittis and Gabaldón 2016a; Vosseberg et al. 2021), which reassure their conclusions.

Among the lineages discussed in these studies, the pair Actinobacteria–Proteobacteria, possesses a higher than average risk of shift (27.78%, Fig. 2c), but with a substantial majority of simulated trees (>70%) still yielding the correct ordering. Additionally, within the 27.78% of trees inducing shifts, 12.7% yield dates that are older than FECA. Therefore, the actual fraction of confounding trees would be around 24%. Consistent with our observations for the ATPase tree, in the alternative LUCA tree we found no conflictive clades among those that have been previously considered in the discussed eukaryogenesis studies (Fig. S2c). Of note, all these simulations contemplated the worst-case scenario where all the transfer events originate from ghost lineages.

To test the impact of the proportion of ghost donors, we computed the fraction of shifted conclusions when the proportion of ghost lineages was different from 1 (Fig. 2d). Our results indicate that the number of inferred shifts increases with higher proportions of ghosts, with a maximum plateau reached when the proportion of ghosts is larger than 80%. It can be expected for the overwhelming majority of lineages alive during FECA–LECA to be extinct now, however the exact percentage of inferred ghosts seems to not play a role in the qualitative conclusions obtained in this analysis.

## Discussion

The comparison of branch lengths across gene trees can provide insights into the relative timing of past evolutionary events. This principle forms the basis of the branch-length ratio method, a technique that has been used to estimate the relative chronology of events during eukaryogenesis (Pittis and Gabaldón 2016a; Vosseberg et al. 2021). However, the accurate inference of these events can be significantly influenced by technical limitations, such as incomplete or unbalanced taxon sampling (Susko et al. 2021; Steenwyk et al. 2023).

Identifying the timeline of gene transfers to the ancestor of eukaryotes is particularly sensitive to the presence and correct identification of donor lineages within sequence databases. Tricou et al. (2022) previously explored the potential impact of unsampled (ghost) lineages using generic tree simulations, which were not specifically tailored to our current understanding of the TOL. In this study, we enhance this approach by using a dated TOL as the phylogenetic backbone where ghost lineages and their transfers to the lineage leading to LECA are simulated. Using this refined framework, we evaluated the proportion of erroneous inferences depending on the phylogenetic placement of the ghost. Despite a non-negligible contribution of “ghost-induced shifts” (averaging around 20%), our findings indicate that a substantial majority of the gene trees would still point to the correct relative ordering of transfer events. This suggests that ghost lineages are unlikely to significantly distort inferences regarding transfers occurring during the FECA–LECA period. Moreover, our simulations demonstrate that the proportion of shifts converges toward the expected values (as inferred from the simulations in Fig. 2a and b) as the proportion of ghost lineages in the tree increases. The fact that the proportion of shifts reaches a plateau and does not continue increasing linearly with the proportion of ghosts, underscores the robustness of our inferred values and suggests they are not merely artifacts of the modeling approach. This trend persists even when an alternative TOL with less comprehensive taxonomic coverage is used, although with a higher incidence of ghost-induced shifts, suggesting an important role of extensive and representative taxonomic sampling in mitigating the effects of ghost lineages. A previous study assessed the effect on the likelihood of shifts of different extinction rates and number of species considered in simulated species trees, concluding that correct inferences were the majority across all conditions and increased with higher extinction rates and larger number of species considered (Bernabeu et al. 2024a). Combined, these studies highlight the relevance of the underlying species tree topology on assessing the impact of ghost lineages, but also underscore that correct conclusions are more likely than incorrect ones across a broad range of conditions.

Notably, a significant fraction of shift-inducing trees (around 20%) would result in ages older than FECA and would typically be disregarded as artifacts in eukaryogenesis studies, thereby further minimizing the impact of shift-inducing ghosts. While the identification of branches longer than FECA ideally requires dated trees, such branches would likely appear as extreme outliers even in undated trees. An interesting avenue for future research would be to identify sets of genes inferred to have been acquired from the same donor at similar relative times. The selection of such consistent “modules” rather than individual gene trees would minimize the risk of wrong inferences, and allow discarding gene trees with outlier branch lengths. A valuable insight from our simulation framework is that it allows us to assess the likelihood of shifts depending on the phylogenetic position of the identified donor. Importantly, the problematic lineages inducing the highest proportions of shifts are outside the currently considered bacterial donors in eukaryogenesis (Pittis and Gabaldón 2016a). Even the clades of interest with the highest estimated shift risks (Proteobacteria and Actinobacteria) present a probability of shifts well below 50% and only slightly higher than the average. This suggests that a large majority of gene trees would still yield correct inferences of relative order.

Altogether, our results suggest that the overall impact of ghost lineages on the specific problem of gene flow during eukaryogenesis is likely limited, supporting the conclusions of earlier studies using the branch-length ratio method (Pittis and Gabaldón 2016a; Vosseberg et al. 2021). In addition, our analyses point toward potential control or mitigating strategies. Firstly, simulations can proactively identify donor clades that are prone to generating misleading results, allowing for careful consideration during data interpretation. Secondly, our findings suggest that discarding gene trees with unusually long branches (as those pointing to transfers before FECA in our simulations) and selecting groups of genes exhibiting similar branch length distributions may effectively reduce the potentially misleading effects of ghosts.

In conclusion, our proposed simulation approach provides greater clarity on the impact of ghost lineages on the relative timing of gene transfer during eukaryogenesis, lending further support to previous inferences. Moreover, it offers strategies for identifying potential artifacts and mitigating the confounding effects of ghost lineages. Beyond the specific study of eukaryotic origins, our approach can be readily adapted to investigate other evolutionary questions.

## Methods

### Phylogeny-aware Simulation Framework

In this simulation framework, we employ an empirical dated species tree, which we populate with simulated ghost lineages that branch out from any branch in the tree that coexist with the relevant recipient lineage. We then simulate gene transfers from these simulated ghost lineages to the recipient lineage. Then, for a given pair of simulated ghost lineages we assess whether the relative ordering inferred in the absence of ghost is reversed with respect to the real order in which the events were simulated, a situation defined as a “shift”.

More formally, this framework can be described as follows: given a dated (or ultrametric) species phylogeny and an acceptor branch representing the lineage that received the gene transfers, we simulate two ghost lineages in each iteration. These two lineages originate from branches in the species phylogeny that are different from the acceptor branch but that overlap in time (i.e. coexist), at least partially, with it, as they need to coexist in time in order to have feasibly participated in HGT. Then, for each simulated ghost we simulate a gene transfer to the acceptor branch at a randomly chosen time at which both ghost and acceptor branches exist. Assuming an inference is subsequently done in the absence of the ghost, the expected inferred donor lineage would correspond to the extant lineage that is closest to the ghost, and the inferred length of the branch corresponding to the transfer will correspond to the time elapsed between the last common ancestor of the acceptor branch and the inferred donor, and the end of the acceptor branch. This allows us to assess differences between the true and the inferred order of gene transfer events, depending on the phylogenetic position of the putative ghost donors. The procedure is repeated for the desired number of iterations, and the species tree, the acceptor branch, and the number of ghost lineages can be varied depending on the scenario to test.

### Testing Gene Flow in Eukaryogenesis

We adapted the above framework to specifically test the effect of ghosts on the scenario relevant for eukaryogenesis (Pittis and Gabaldón 2016a; Vosseberg et al. 2021). For this, we used as a species tree a recently inferred dated phylogenetic TOL obtained from a concatenation of the ribosomal markers for the ATPase distribution analysis (Mahendrarajah et al. 2023) (the ATPase tree, Fig. 1a). We removed the chloroplast and mitochondria clades to obtain a reliable set of putative bacterial sisters for the ghost donor. We set the acceptor branch to be the branch connecting the last common ancestor of eukaryotes and their closest Asgardarchaeota relatives (which, for simplicity, we here name the FECA, and the LECA, the FECA–LECA lineage (Gabaldón 2021). We dated FECA and LECA using the tree branches (i.e. the mean of the posterior distribution of the node datings), obtaining a FECA–LECA time frame of 2.42 to 1.89 Ga (Fig. 1b). Finally, we retrieved the birth and death of the branches present in the inferred FECA–LECA period, which we called the FECA–LECA branch space (Fig. S4b). The simulated ghost lineages had to comply with the following conditions: (i) they branch out from a bacterial lineage (i.e. they are bacterial); (ii) they must have branched out from a branch that coexisted with the FECA–LECA branch; (iii) the transfer from the donor ghost lineage had to have occurred during the FECA–LECA period; (iv) the death of the ghost branch had to occur after the transfer, at any point up to the present (Fig. 1c). Note that this last point is a formalism and does not take part in the assessment of shifts. At the end of the simulation, we calculated the proportion of iterations in which the early/late donor classification (i.e. the relative ordering of the transfers) is reversed if the lineages are unsampled, that is, when there is a “shift” in the relative ordering of the observed transfers. This process is agnostic to whether the lineage is extinct (its death occurred in the past) or just unsampled (data on its existence is still missing), therefore covering the range of scenarios by which a lineage may be a ghost.

To simulate transfers from ghost lineages to the proto-eukaryote under the aforementioned premises, we used the following process: (i) we randomly selected a branch from the FECA–LECA branch space (Fig. S4), which is then considered the extant sister of the ghosts. (ii) The birth and transfer events of the ghost lineage are inferred depending on the birth and death of the selected (sister) branch: If the branch exists during the whole FECA–LECA period, the ghost birth is calculated between the birth and LECA, and the ghost transfer to the FECA–LECA branch is inferred in the period between the ghost birth and LECA; if the branch originated before FECA and died in the FECA–LECA period, the ghost birth is inferred from the birth and the death of the branch, the transfer is then sampled in the segment of the branch that falls in the FECA–LECA period; if the ghost birth is inferred before FECA, we sampled the transfer from FECA to LECA, whereas when the ghost birth is sampled inside the FECA–LECA period, we inferred the transfer in the period between the ghost birth and LECA; and if the branch originated after FECA and died after LECA, we inferred the ghost birth between the branch birth and LECA, and the transfer between the ghost birth and LECA.

### Conclusion Shift Test

In a simulation round, we generated 1,000 pairs of transfers from simulated ghosts, with each pair of transfers comprising an “early” and a “late” transfer, depending on their relative distance to the LECA node. We then retrieved the date of the birth of the extant sister clade from within the simulated ghost branches, that is, the transfer date which we would infer in the gene tree. Although this inferred date does not correspond to the actual date of the transfer, it is the only measurable branch length and the proxy used in the branch-length ratio method to approximate the age of the transfer, as done in previous studies. The birth dates of the sisters were then also classified as “early” and “late” with respect to the LECA node (indicating which event occurred before and which after in the timeline of events), and we, finally, assessed whether the births of the sister clades and the actual transfers had the same order or, otherwise, resulted in a shift, in the latter case, we called a conclusion shift. This simulation round was then repeated 1,000 times (a total of 1,000,000 transfer pairs), allowing us to get a distribution of the percentage of conclusion shifts and the number of branch lengths that, once shifted, result in distances longer than FECA (and that therefore would be considered aberrant and discarded from analysis).

### Impact of the Proportion of Ghost Lineages

To assess the impact of the proportion of ghost lineages in the tree, we simulated pairs of transfers in which we assigned them to be ghosts or not. For each transfer pair, we computed an observed age for the transfer. We first determined whether the donor clade is extant or a ghost following a Bernoulli distribution with a given probability (the proportion of ghost lineages). The observed age is assigned to the transfer age (if the donor is inferred to be alive) or to the ghost birth age (in the case of a ghost donor). Each transfer event is treated as independent, opening the possibility to obtain combinations of nonghost and ghost donors, which are of interest, as they can provide larger differences between observed distances. We then compared the early–late conclusion in both the observed ages and the transfer ages to test whether the conclusions shifted.

We implemented this simulation procedure in R, the code for these functions is stored in the GitHub repository for the project https://github.com/Gabaldonlab/ghost_transfers.

## Supplementary Material

evaf190_Supplementary_Data

## Data Availability

This study has not generated new data, the code to obtain the simulations is available in https://github.com/Gabaldonlab/ghost_transfers. The reference trees are deposited in the respective repositories of the papers: 10.5281/zenodo.10012837 for the ATP-synthase tree, and 10.6084/m9.figshare.24428659 for the LUCA tree.
