# Supplementary material for: Phylogeny-aware Simulations Suggest a Low Impact of Unsampled Lineages in the Inference of Gene Flow During Eukaryogenesis
Source: Genome Biol Evol. 2025 Oct 9;17(11):evaf190. doi: 10.1093/gbe/evaf190 (PMC12573248; doi:10.1093/gbe/evaf190)
Supplement: evaf190_Supplementary_Data [file evaf190_supplementary_data.pdf]

Supplementary Material for: Phylogeny-aware simulations suggest a low impact of unsampled lineages in the inference of gene flow during eukaryogenesis

Moisès Bernabeu[1,2,+], Saioa Manzano-Morales[1,2,+], Toni Gabaldón[1,2,3,4*]

[1] Barcelona Supercomputing Centre (BSC-CNS) Plaça Eusebi Güell, 1-3, 08034, Barcelona, Spain.
[2] Institute for Research in Biomedicine (IRB Barcelona), The Barcelona Institute of Science and Technology, Baldiri Reixac, 10, 08028, Barcelona, Spain.
[3] Catalan Institution for Research and Advanced Studies (ICREA), Barcelona, Spain.
[4] Centro de Investigación Biomédica En Red de Enfermedades Infecciosas (CIBERINFEC), Barcelona, Spain.
+ These authors contributed equally to the work

*Corresponding author: toni.gabaldon@bsc.es

Supplementary materials.......... 2
Supplementary Figures.......... 2
Supplementary Tables.......... 5
Supplementary References.......... 10

Supplementary materials

Supplementary Figures

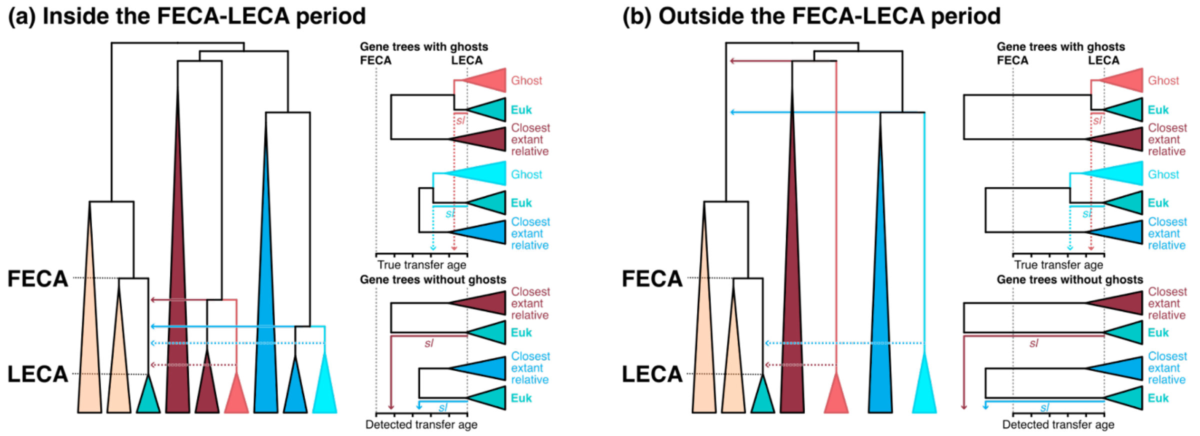

Supplementary Fig. S1. Conditions to detect ages older than FECA. a) As in Fig. 2a, the topology of the dated TOL and the expected gene trees when ghosts are in the tree or absent. Dashed lines show the transfer ages, whereas the solid lines are the detected ages when the ghost lineage is absent. b) Necessary conditions to detect transfer ages outside the FECA-LECA period. The divergence of the ghost clade that transferred to the protoeukaryote and its extant sister occurred before FECA. Therefore, the detected transfer ages, which are shifted, would be detected outside the FECA-LECA period and, thus, we could remove them.

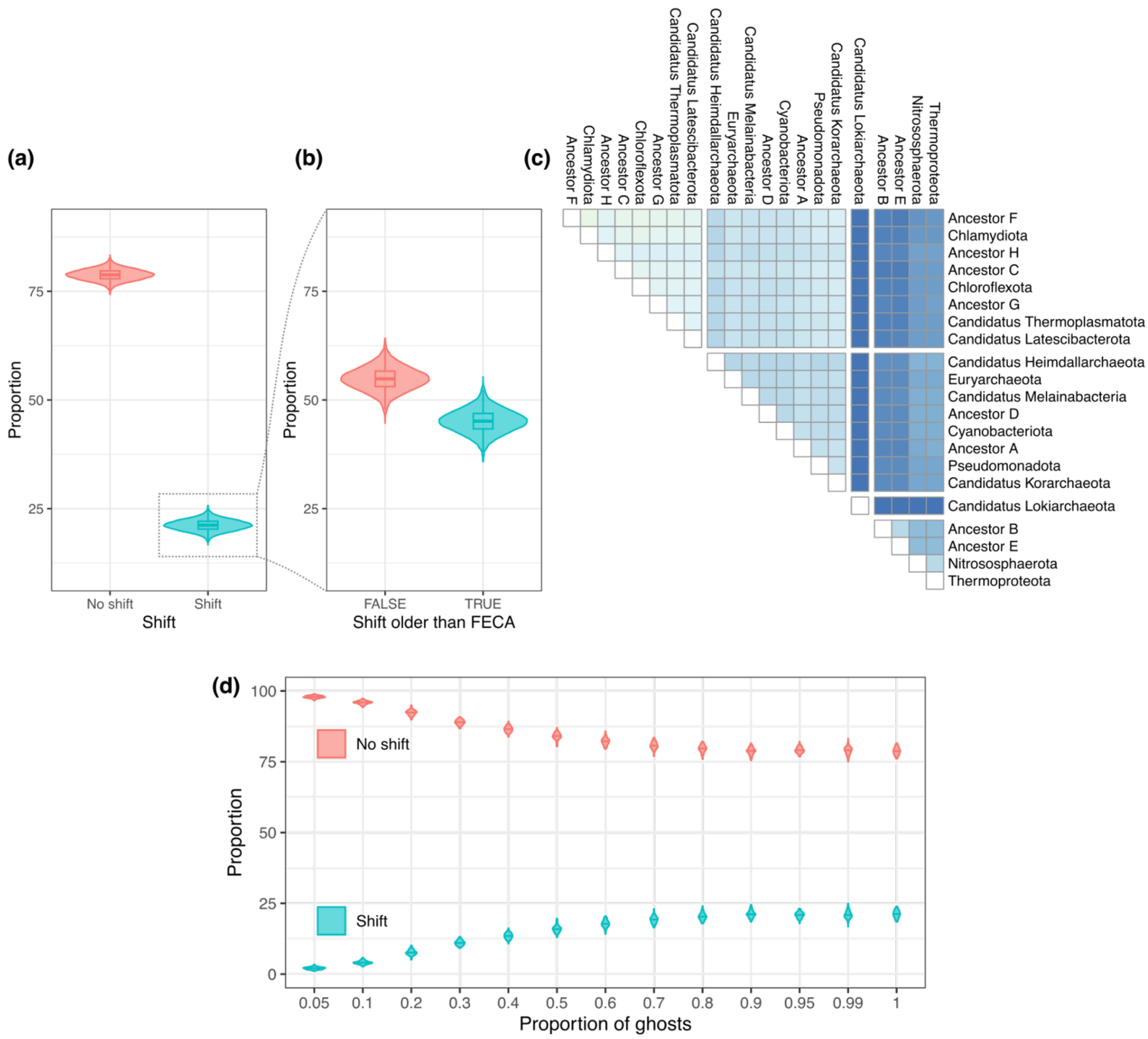

Supplementary Fig. S2. Proportion of shifted conclusions for the tree from Moody et al. (2024). a) Distributions for the proportion of shifts observed between pairs of simulated ghosts (left). b) For the shift-inducing cases, proportion of those that show an inferred distance older than FECA (outside FECA-LECA period). b) Proportion of shifts per pair of lineages, that is the proportion of simulations resulting in a shift in transfers from the specified pair ancestors. Pseudomonadota contains the proteobacterial clades. The ancestors and their descendant phyla are associated in Supplementary Table S2. d) Proportion of shifts observed between pairs of simulated transfers for a given proportion of ghost lineages. The transfer donors are inferred to be or not a ghost using a Bernoulli distribution with the probability parameter specified in the x-axis.

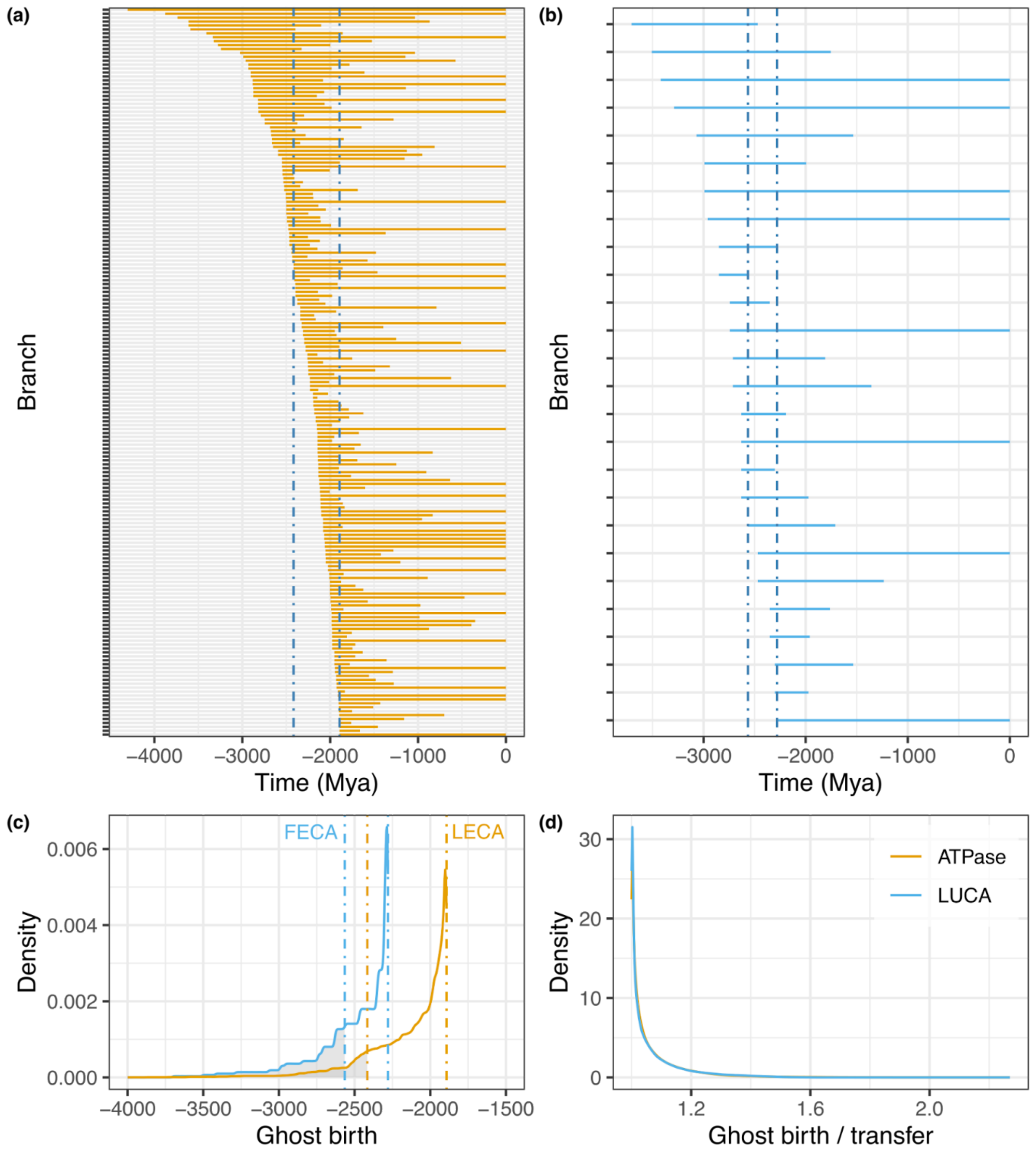

Supplementary Fig. S3. The effect of sampling in the branch space. a) ATPase tree (Mahendrarajah et al. 2023) branch space coexisting with LECA. b) LUCA tree (Moody et al. 2024) branch space coexisting with LECA. c) Distribution of the birth of the simulated ghosts, the vertical dashed lines show the FECA and LECA estimates for each tree, and the shadowed area is the proportion of ghost births older than FECA. d) Ratio between the ghost birth and the transfer ages. The colours of the line in all the panels show the trees used following the legend in panel d).

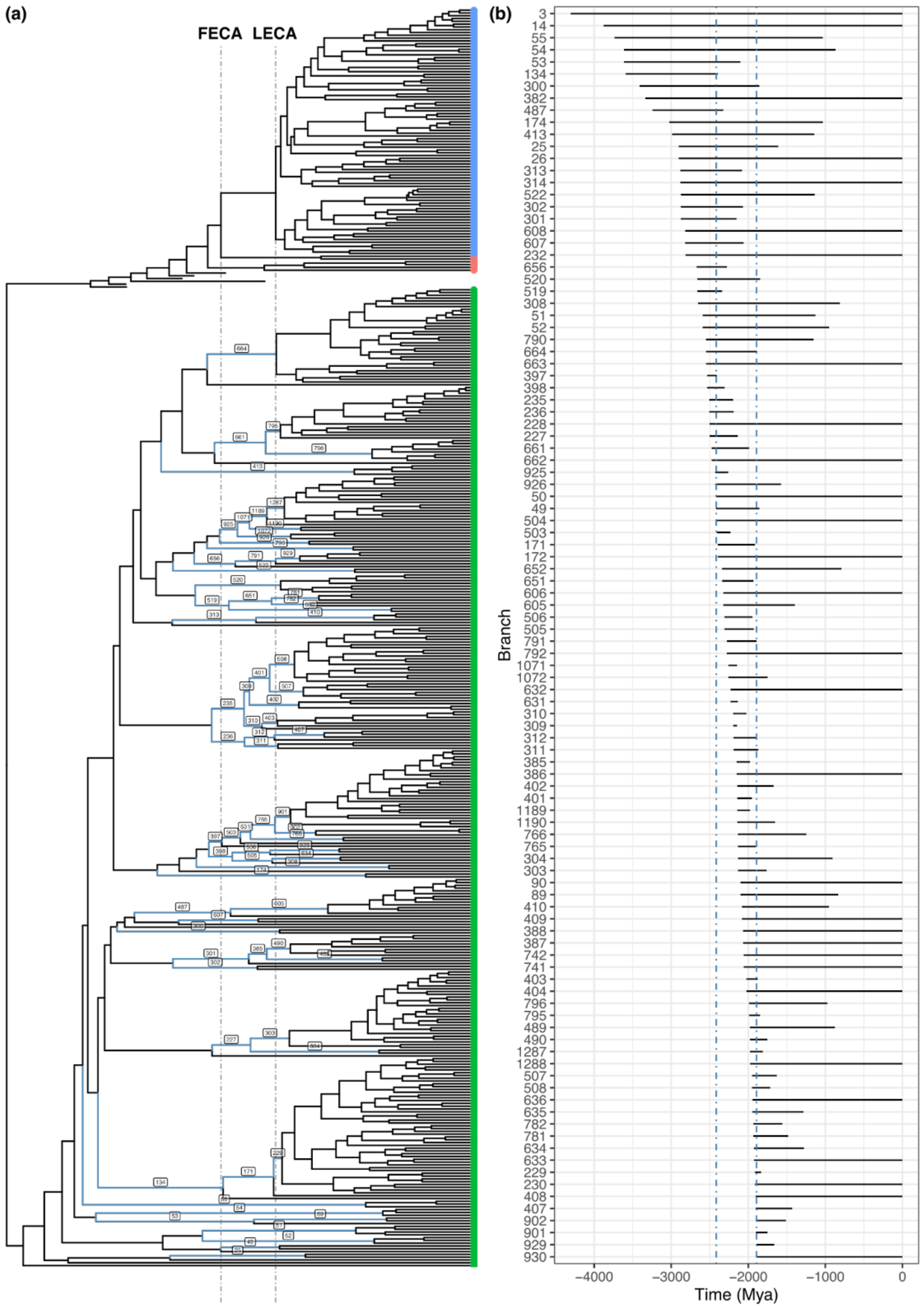

Supplementary Fig. S4. The branch space. a) Dated phylogeny from Mahendrarajah et al. (2023) with the branches present in the FECA-LECA period in blue, the branch label corresponds to the number of the branch. Split branches correspond to archaeal lineages different from Asgardarchaeota. b) The set of branches present in the FECA-LECA period sorted by birth date, the number corresponds to the number of the branch in a).

Supplementary Tables

Supplementary Table S1. Correspondence between ancestral donor nodes and their extant daughter phyla. The none clade refers to a set of clades that do not have an associated phylum, as the taxonomy is inherited from the original tree in Mahendrarajah et al. (2023).

Ancestor	Members
**Ancestor AA**	Lentisphaerae
	Kiritimatiellaeota
**Ancestor AB**	Rhodothermaeota
	Balneolaeota
	Bacteroidetes
	Ignavibacteriae
	none
	Chlorobi
	Candidatus
**Ancestor AC**	DPANN
	Euryarchaeota
**Ancestor AD**	Kiritimatiellaeota
	Lentisphaerae
	Verrucomicrobia
**Ancestor AE**	Tenericutes
	Firmicutes
**Ancestor AF**	Euryarchaeota
	TACK
**Ancestor AG**	TACK
	Archaea
**Ancestor AH**	Candidatus
	Gemmatimonadetes
**Ancestor AI**	candidate
	Candidatus
**Ancestor AJ**	Fibrobacteres
	Candidatus
**Ancestor AK**	none
	Candidatus
**Ancestor AL**	Kiritimatiellaeota

	Chlamydiae
	Lentisphaerae
	Verrucomicrobia
**Ancestor AM**	Fibrobacteres
	candidate
	Gemmatimonadetes
	Candidatus
**Ancestor AN**	Elusimicrobia
	Candidatus
**Ancestor AO**	Proteobacteria
	Acidobacteria
	Candidatus
**Ancestor AP**	Chrysiogenetes
	Nitrospinae
	Deferribacteres
	Candidatus
**Ancestor AQ**	Rhodothermaeota
	Balneolaeota
	Bacteroidetes
	Ignavibacteriae
	Chlorobi
	Candidatus
**Ancestor AR**	Proteobacteria
	Nitrospinae
	Nitrospirae
	Chrysiogenetes
	Acidobacteria
	Deferribacteres
	Thermodesulfobacteria
	Candidatus
**Ancestor AS**	Fibrobacteres
	candidate
	Candidatus

**Ancestor AT**	TACK
	Euryarchaeota
**Ancestor AU**	Coprothermobacterota
	Caldiserica
**Ancestor AV**	Ignavibacteriae
	Candidatus
**Ancestor AW**	Proteobacteria
	Nitrospinae
	Chrysiogenetes
	Deferribacteres
	Thermodesulfobacteria
	Candidatus
**Ancestor AX**	Proteobacteria
	Nitrospinae
	Nitrospirae
	Chrysiogenetes
	Deferribacteres
	Thermodesulfobacteria
	Candidatus
**Ancestor AY**	Proteobacteria
	Thermodesulfobacteria
**Ancestor AZ**	Rhodothermaeota
	Chlorobi
	Balneolaeota
	Bacteroidetes
**Ancestor BA**	Acidobacteria
	Candidatus

Supplementary Table S2. Correspondence between ancestral donor nodes and their extant daughter phyla. The taxonomy is inherited from the original tree in Moody et al. (2024).

Ancestor	Members
**Ancestor A**	Chlorobiota
	Candidatus Cloacimonadota
	Elusimicrobiota
	Calditrichota
	Verrucomicrobiota
	Synergistota
	Lentisphaerota
	Dictyoglomota
	Caldisericota
	Planctomycetota
	Campylobacterota
	Thermotogota
	Fibrobacterota
**Ancestor B**	Chlorobiota
	Elusimicrobiota
	Calditrichota
	Verrucomicrobiota
	Synergistota
	Lentisphaerota
	Planctomycetota
	Campylobacterota
	Candidatus Cloacimonadota
	Fibrobacterota
**Ancestor C**	Nitrososphaerota
	Thermoproteota
**Ancestor D**	Acidobacteriota
	Nitrospirota
	Thermodesulfobacteriota
	Aquificota
**Ancestor E**	Thermotogota
	Dictyoglomota

	Caldisericota
**Ancestor F**	Bacillota
	Actinomycetota
**Ancestor G**	Euryarchaeota
	Candidatus Thermoplasmatota
**Ancestor H**	Candidatus Thorarchaeota
	Candidatus Lokiarchaeota
	Candidatus Odinarchaeota

Supplementary References

Mahendrarajah TA et al. 2023. ATP synthase evolution on a cross-braced dated tree of life. Nat Commun. 14:7456. doi: 10.1038/s41467-023-42924-w.

Moody ERR et al. 2024. The nature of the last universal common ancestor and its impact on the early Earth system. Nat Ecol Evol. 8:1654–1666. doi: 10.1038/s41559-024-02461-1.
